# Complete Genome Sequence of Industrial Biocontrol Strain *Paenibacillus polymyxa* HY96-2 and Further Analysis of Its Biocontrol Mechanism

**DOI:** 10.3389/fmicb.2018.01520

**Published:** 2018-07-12

**Authors:** Yuanchan Luo, Yuejuan Cheng, Jincui Yi, Zhijun Zhang, Qian Luo, Daojing Zhang, Yuanguang Li

**Affiliations:** State Key Laboratory of Bioreactor Engineering, East China University of Science and Technology, Shanghai, China

**Keywords:** genome sequencing, *Paenibacillus polymyxa*, comparative genomic analysis, biocontrol mechanism, biofilm, antibiotics, induced resistance

## Abstract

*Paenibacillus polymyxa* (formerly known as *Bacillus polymyxa*) has been extensively studied for agricultural applications as a plant-growth-promoting rhizobacterium and is also an important biocontrol agent. Our team has developed the *P. polymyxa* strain HY96-2 from the tomato rhizosphere as the first microbial biopesticide based on *P. polymyxa* for controlling plant diseases around the world, leading to the commercialization of this microbial biopesticide in China. However, further research is essential for understanding its precise biocontrol mechanisms. In this paper, we report the complete genome sequence of HY96-2 and the results of a comparative genomic analysis between different *P. polymyxa* strains. The complete genome size of HY96-2 was found to be 5.75 Mb and 5207 coding sequences were predicted. HY96-2 was compared with seven other *P. polymyxa* strains for which complete genome sequences have been published, using phylogenetic tree, pan-genome, and nucleic acid co-linearity analysis. In addition, the genes and gene clusters involved in biofilm formation, antibiotic synthesis, and systemic resistance inducer production were compared between strain HY96-2 and two other strains, namely, SC2 and E681. The results revealed that all three of the *P. polymyxa* strains have the ability to control plant diseases via the mechanisms of colonization (biofilm formation), antagonism (antibiotic production), and induced resistance (systemic resistance inducer production). However, the variation of the corresponding genes or gene clusters between the three strains may lead to different antimicrobial spectra and biocontrol efficacies. Two possible pathways of biofilm formation in *P. polymyxa* were reported for the first time after searching the KEGG database. This study provides a scientific basis for the further optimization of the field applications and quality standards of industrial microbial biopesticides based on HY96-2. It may also serve as a reference for studying the differences in antimicrobial spectra and biocontrol capability between different biocontrol agents.

## Introduction

*Paenibacillus polymyxa* is a multifunctional Gram-positive (G^+^) bacterium that has been reported to have applications in agriculture (Hao and Chen, 2017), medicine (Galea et al., 2017; Yu et al., 2017), and industry (Lal and Tabacchioni, 2009). *P. polymyxa* has been in the list of substances reported under the Toxic Substance Control Act (TSCA) by the United States Environmental Protection Agency (EPA)^1^. It means *P. polymyxa* is a safe and commercially available microbe. The application of *P. polymyxa* in agriculture includes two main aspects: the biocontrol of plant diseases and the promotion of plant growth. *P. polymyxa* is an important biocontrol agent that has been reported to suppress a large variety of fungal and bacterial plant diseases, such as those caused by the fungi *Fusarium oxysporum* and *Botrytis cinerea* and the bacteria *Xanthomonas campestris* and *Ralstonia solanacearum* (Xu et al., 2006; Kim et al., 2010; Fan et al., 2012; Weselowski et al., 2016). *P. polymyxa* can withstand various adverse conditions during the biopesticide manufacturing process, especially drying, and exhibits a long and resilient shelf life owing to its endogenous spore formation. Therefore, *P. polymyxa* is an important strain for the production of microbial biopesticides^2^^,^^3^. In 2004, *P. polymyxa* strain HY96-2 from the tomato rhizosphere was developed as the first microbial biopesticide based on *P. polymyxa* and registered in China, for the control of soil-borne diseases caused by *R. solanacearum* on tomatoes and *F. oxysporum* on watermelons, as well as leaf diseases caused by *B. cinerea* and *Pseudomonas syringae* on cucumbers^2^. In addition, other studies have demonstrated that HY96-2 inhibits the growth of many fungal and bacterial pathogens, such as *Colletotrichum gloeosporioides*, *Rhizoctonia solani*, and *Erwinia carotovora* (Fan et al., 2012). Although microbial biopesticides derived from *P. polymyxa* HY96-2 have been manufactured and sold in 24 provinces across China, further research into its precise biocontrol mechanism, especially at the molecular level, is still needed.

Genomes are a very useful resource for understanding the mechanism of biocontrol agents. Kim et al. (2017) determined the key genes responsible for the production of antimicrobial agents and volatile organic compounds, indoleacetic acid (IAA) synthesis, siderophore secretion, phosphate transporter, and phosphonate cluster biosynthesis in *Paenibacillus yonginensis* DCY84T by genome sequencing and confirmed the ability of this strain to induce plant resistance and protect plant growth by a combination of physiological experiments. By comparing the genomes of *Bacillus amyloliquefaciens* FZB42 with *B. subtilis*, Chen et al. (2007) discovered that over 8.5% of the genome of *B. amyloliquefaciens* FZB42 is involved in antibiotic and siderophore synthesis, whereas Stein (2005) estimated that not more than 4–5% of the average *B. subtilis* genome is devoted to antibiotic production. Therefore, this genomic comparison demonstrated that *B. amyloliquefaciens* FZB42 is capable of protecting plants from diseases at the molecular level via the production of antibiotics.

Furthermore, despite its importance as a biocontrol agent, there have only been a small number of comprehensive studies into the biocontrol mechanism of *P. polymyxa* using genomic comparisons or other molecular methods. To further understand the biocontrol mechanism of HY96-2 at the molecular level, its genome was completely sequenced and compared with those of other *P. polymyxa* strains. Thus far, the complete genomes of seven *P. polymyxa* strains have been published, including the strains SC2 (Ma et al., 2011), E681 (Kim et al., 2010), YC0136 (Liu et al., 2017a), M-1 (Niu et al., 2011), SQR-21 (Li et al., 2014), CR1 (Eastman et al., 2014b), and YC0573 (Liu et al., 2017b). The details of these strains are presented in **Table 1**. Compared with other *P. polymyxa* strains, SC2 and E681 have been studied in greater detail and found to inhibit the growth of many plant pathogens. SC2 was isolated from the rhizosphere of pepper plants in Guizhou Province, China, and demonstrated to inhibit plant pathogenic fungi, such as *F. oxysporum*, *B. cinerea*, *Pseudoperonospora cubensis*, and plant pathogenic bacteria, the species of which were not published (Zhu et al., 2008). E681, isolated from the rhizosphere of barley plants in South Korea, was reported to possess inhibitory activity against plant pathogenic fungi such as *F. oxysporum*, *B. cinerea*, and *R. solani* (Ryu et al., 2006) and the plant pathogenic bacterium *P. syringae* (Kwon et al., 2016).

**Table 1:** Sources and functions of the studied *P. polymyxa* strains (the complete genomes of which have been published).

Name	Accession number(s)	Origin	Activity	Reference
HY96-2	CP025957	Tomato rhizosphere (Nanchang, Jiangxi, China)	As biocontrol agent against fungi (*F. oxysporum*, *C. gloeosporioides*, *B. cinerea*, *R. solani*, *Alternaria solani*, *Penicillium italicum*, *Curvularia lunata*, etc.) and Gram-negative (G^-^) bacteria (*R. solanacearum*, *P. syringae*, *E. carotovora*, etc.).	Industrial microbial biopesticides that have been commercially registered in China; Fan et al., 2012
SC2	CP002213- CP002214	Pepper rhizosphere (Guizhou, China)	As biocontrol agent against fungi (*F. oxysporum*, *B. cinerea*, and *P. cubensis*) and bacteria (species not stated), and as plant-growth-promoting rhizobacterium (PGPR) to dissolve phosphorus and potassium.	Zhu et al., 2008; Ma et al., 2011
E681	CP000154	Winter barley rhizosphere (South Korea)	As biocontrol agent against fungi (*F. oxysporum*, *B. cinerea*, *B. allii, R. solani*, *Pythium* sp., *Aspergillus* sp., etc.), and bacteria (*P. syringae*, *E. coli*, etc.), and as PGPR.	Ryu et al., 2006; Kim et al., 2010; Kwon et al., 2016
YC0136	CP017967	Tobacco rhizosphere (Guizhou, China)	As biocontrol agent against fungus *Phytophthora parasitica* var. *nicotianae* and as PGPR.	Liu et al., 2017a
M-1	HE577054- HE577055	Wheat rhizosphere (China)	As biocontrol agent against fungus *R. solani* and as PGPR.	Niu et al., 2011
SQR-21	CP006872	Melon rhizosphere (China)	As biocontrol agent against fungus *F. oxysporum* and as PGPR.	Yaoyao et al., 2017
CR1	CP006941	Degraded corn straw (Southern Ontario, Canada)	As biocontrol agent to antagonize fungi (*R. solani*, *Cylindrocarpon destructans*, etc.) and bacteria (*P. syringae*, *X. campestris*, etc.), and as PGPR, as well as biodegradation agent.	Eastman et al., 2014b; Weselowski et al., 2016
YC0573	CP017968	Tobacco rhizosphere (Guizhou, China)	As biocontrol agent to antagonize fungus *P. parasitica* var. *nicotianae* and as PGPR.	Liu et al., 2017b


Colonization (Bianciotto et al., 2001; Bais et al., 2004; Sang and Kim, 2014), antagonism (Lugtenberg and Kamilova, 2009; Sharma et al., 2009), and induced resistance (Ongena et al., 2007; Shi et al., 2017) are the three main reported mechanisms of biological control. Biofilm formation indicates that a biocontrol agent possesses good colonization ability (Ongena and Jacques, 2008; Sang and Kim, 2014). Besides the key genes involved in biofilm formation, quorum sensing also plays an important role (Miller and Bassler, 2001). The types and amounts of antibiotics generated by a biocontrol agent affect their antimicrobial spectra and biocontrol efficacies. It has been reported that *P. polymyxa* secretes antifungal and antibacterial metabolites, which mainly include fusaricidins, polymyxins, and other antibiotics (Jeong et al., 2011; Shaheen et al., 2011). Biocontrol agents can also generate and release systemic resistance inducers including volatile organic compounds (mainly consisting of 2,3-butanediol, methanethiol, isoprene, butyl acetate, *n*-hexadecane, etc.) into the surrounding environment, both of which have been shown to have a positive effect on plant protection (Ryu et al., 2004; Lee et al., 2012; Shi et al., 2017).

To the best of our knowledge, no genomic comparison has yet been applied to analyze the biocontrol mechanism of *P. polymyxa*. Thus, in this study the complete genome of *P. polymyxa* strain HY96-2 was sequenced and compared with those of seven other strains. To elucidate the differences in the biocontrol mechanisms between strain HY96-2 and other agriculturally undeveloped *P. polymyxa* strains, the main genes (or gene clusters) of HY96-2 involved in biofilm formation, antibiotic synthesis, and systemic resistance inducer production were analyzed in comparison with the strains SC2 and E681. This study provides a scientific basis for the further optimization of the field applications and product quality standards of the microbial biopesticide derived from *P. polymyxa* HY96-2.

## Materials and Methods

### Strains and Genomic DNA Preparation

*P. polymyxa* strain HY96-2 was isolated from the rhizosphere of tomato plants in the suburbs of Nanchang, Jiangxi Province, China, and preserved in the China General Microbiological Culture Collection Center (CGMCC No. 0829). HY96-2 was cultured at 30°C in Luria–Bertani broth. Genomic DNA was purified from overnight liquid cultures (OD_600 nm_ ≈ 0.7) using the cetyltrimethylammonium bromide method (Watanabe et al., 2010). A TBS-380 fluorometer (Turner BioSystems, United States) or NanoDrop 2500 (Thermo Scientific, United States) was applied to ensure the DNA quality (≥10 μg, without degradation, OD_260_/OD_280_ ≈ 1.8–2.0).

### Sequencing and Assembly

The whole genome was sequenced using the PacBio RS II platform with a 10-kb library. Reads were assembled using HGAP (version 2.3.0, SMRT Analysis) (Chin et al., 2013). The assembly data for the complete genome have been deposited in GenBank with the accession number CP025957.

### Genome Components and Genome Annotation

Coding DNA sequence (CDS) prediction was performed using Glimmer 3.02 (Delcher et al., 1999). A circular map of the genome was obtained using Circos version 0.64 (Krzywinski et al., 2009). Genomic islands (GIs) were predicted using the GI prediction method IslandViewer 4 (Dhillon et al., 2015). tRNA and rRNA were predicted using the tRNAscan-SEv1.3.1 (Lowe and Eddy, 1997) and barrnap 0.7 software^4^, respectively. Clustered regularly interspaced short palindromic repeat sequences (CRISPRs) were found using CRISPRFinder (Grissa et al., 2007). Functional annotation was based on BLASTP searches (BLAST 2.2.28+) against the NCBI non-redundant (NR) database, gene database, string database, and gene ontology (GO) database. Based on the string database, the BLASTP comparison was used to perform the Clusters of Orthologous Groups of proteins (COG) annotation, according to which the protein function could be classified (Tatusov et al., 2001). The BLAST algorithm was used to compare the predicted genes with the KEGG database, and the corresponding genes involved in specific biological pathways could be obtained according to the KEGG Orthology (KO) numbers obtained from the alignment. GO was annotated with blast2go (Conesa et al., 2005).

### Genome Comparison

The complete genome sequences of seven *P. polymyxa* strains (SC2, E681, YC0136, M-1, SQR-21, CR1, and YC0573) and the related species strain *B. subtilis* 168 (AL009126) and *B. amyloliquefaciens* FZB42 (CP00560) examined in this study were obtained from GenBank. Phylogenetic analysis was conducted for *P. polymyxa* strains, *B. subtilis*, and *B. amyloliquefaciens* inferred by analyzing homologous gene. The single-copy homologous genes of each strain were selected for multiple sequence alignment and quality control comparison. Multiple sequence alignment was conducted by MAFFT software^5^. Quality control comparison was conducted by Gblocks software^6^. Then the phylogenetic tree was constructed by RAxML software with maximum likelihood method based on single gene or multiple genes (Stamatakis, 2014). Pan-genome analysis and nucleic acid co-linearity were conducted for the seven *P. polymyxa* strains and HY96-2. The pan-genome analysis was performed using the OrthoMCL software (Chen et al., 2006). The nucleic acid co-linearity was determined using the MUMmer 3.0 software (Kurtz et al., 2004).

The genes or gene clusters involved in biofilm formation, antibiotic synthesis, and systemic resistance inducer production were compared between strain HY96-2 and strains SC2 and E681. According to the biofilm formation pathways retrieved from the KEGG database^7^, the possible pathways for biofilm formation in *P. polymyxa* were analyzed. In this study, the genes analyzed for biofilm formation in *P. polymyxa* were selected according to Molinatto et al. (2017). The gene clusters for secondary metabolites (containing antibiotics) in *P. polymyxa* were annotated using the antiSMASH database version 4.0.2 and the other antibiotics were selected based on previous studies (Walsh, 2004; Ma et al., 2011). The resistance inducers of *P. polymyxa* in this study were selected according to Lee et al. (2012) and Shi et al. (2017). The key genes involved in resistance inducer synthesis were searched for in the KEGG database. BLAST was used to compare the identities in the genes or gene clusters between HY96-2, SC2, and E681.

## Results

### Genome Features

**FIGURE 1:**
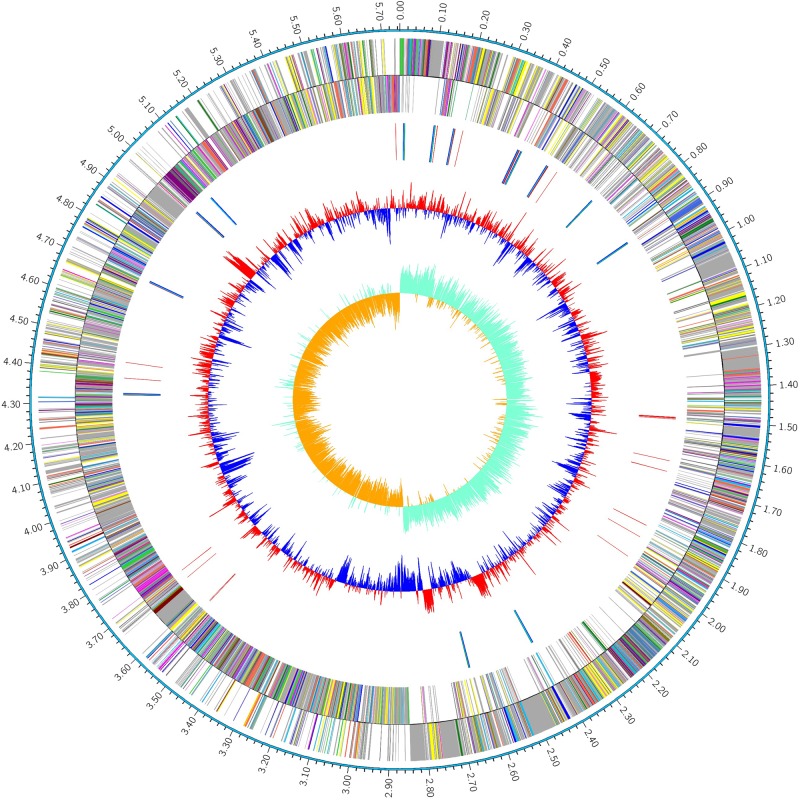
Circular map of the genome of the *P. polymyxa* strain HY96-2. The distribution of the circle from the outside to the inside indicates the genome size, forward CDS, reverse CDS, repeat sequences, tRNA (blue), rRNA (purple), GC ratio (red and blue indicate regions where the GC ratio is higher than average and lower than average, respectively), and GC skew (cyan and orange indicate regions where the G content is greater than and less than the C content, respectively).

The complete genome of *P. polymyxa* strain HY96-2 was 5.75 Mb (**Figure 1**), in which the average GC content of the chromosome was 45.61%. A total of 5207 CDSs were predicted. Furthermore, the HY96-2 genome contained 42 rRNA and 110 tRNA genes. The general features are shown in **Table 2**. However, no plasmids were found using Webcutter version 2.0 or PlasmidFinder version 1.3.

**Table 2:** General features of the genome of the *P. polymyxa* strain HY96-2.

Attribute	Value
Genome size (bp)	5745779
G+C ratio (%)	45.61
Protein-coding genes	5207
Gene total length (bp)	4958220
rRNA	42
tRNA	110
Genes assigned to COGs	2605
Genomic islands	16
CRISPR	1


### Genomic Islands and CRISPR Prediction

Genomic islands often carry genes important for genome evolution and adaptation to surrounding environment such as those involved in pathogenesis and antibiotic resistance. Therefore, GI prediction has gradually become an increasingly important aspect of microbial genome analysis (Lu and Leong, 2016). The GIs predicted in HY96-2 are listed in Supplementary Table S1; a total of 16 GIs were predicted in the chromosome. The CRISPRs containing multiple short and repeated sequences can confer resistance to exogenous genetic elements such as phages and plasmids (Didovyk et al., 2016; Ran, 2017). A putative CRISPR was detected in the HY96-2 genome by CRISPRFinder (Supplementary Table S2).

### Genome Annotation

**FIGURE 2:**
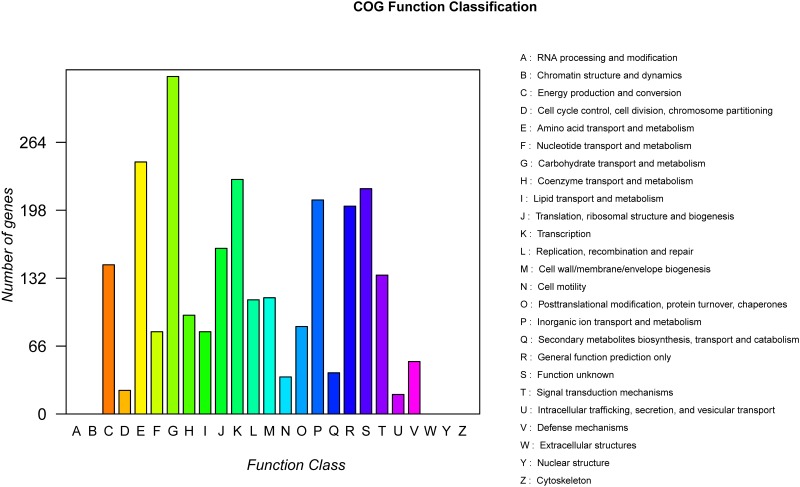
Distribution of genes across COG functional categories in the chromosome of *P. polymyxa* strain HY96-2.

According to the results of the COG annotation, 2605 proteins were classified into 20 COG families (Supplementary Table S3). The largest group of genes was involved in carbohydrate transport and metabolism (328 genes, 6.30%) (**Figure 2**). In total, KEGG orthologs were found for 2352 proteins by BLAST. In addition, the GO annotation results revealed that the largest group of genes was involved in the biological process domain. Among these genes, the content of genes related to the metabolic process category was highest (Supplementary Figure S1).

### Genome Comparison

**FIGURE 3:**
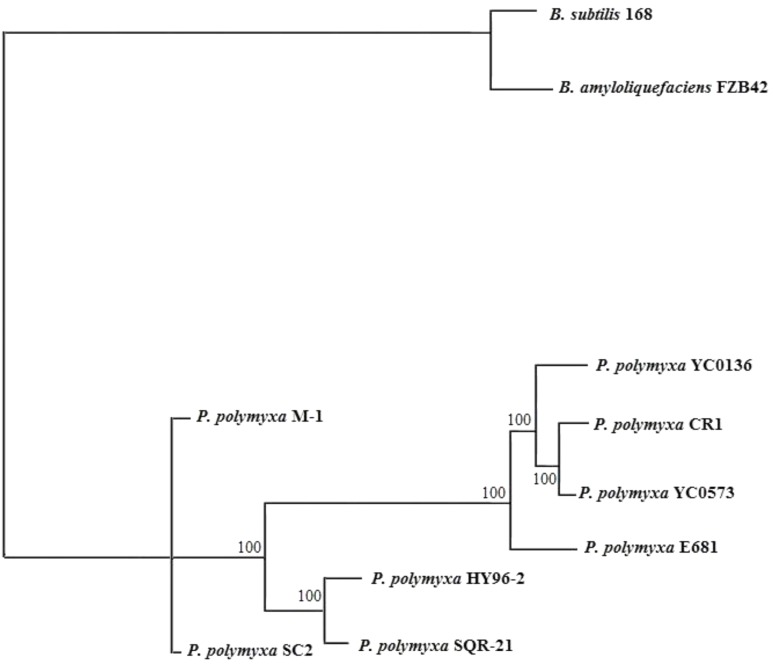
Phylogenetic tree for *P. polymyxa* HY96-2 and the seven other *P. polymyxa* strains, *B. subtilis* 168, and *B. amyloliquefaciens* FZB42 based on homologous genes.

**FIGURE 4:**
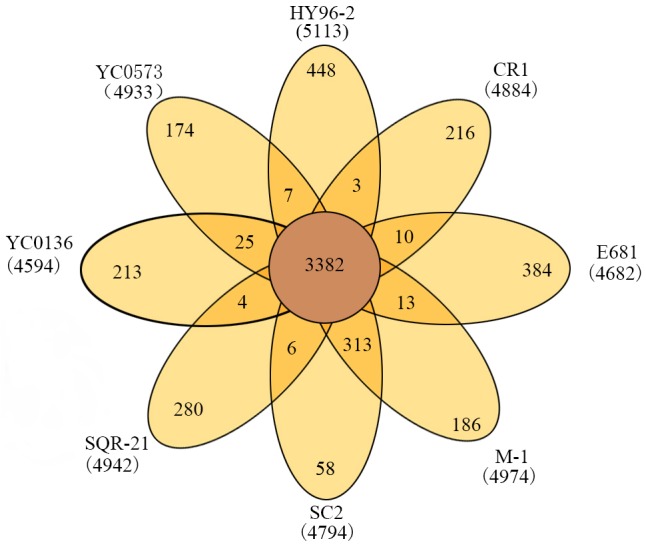
Venn diagram showing numbers of specific and shared gene families among the eight different *P. polymyxa* strains.

**FIGURE 5:**
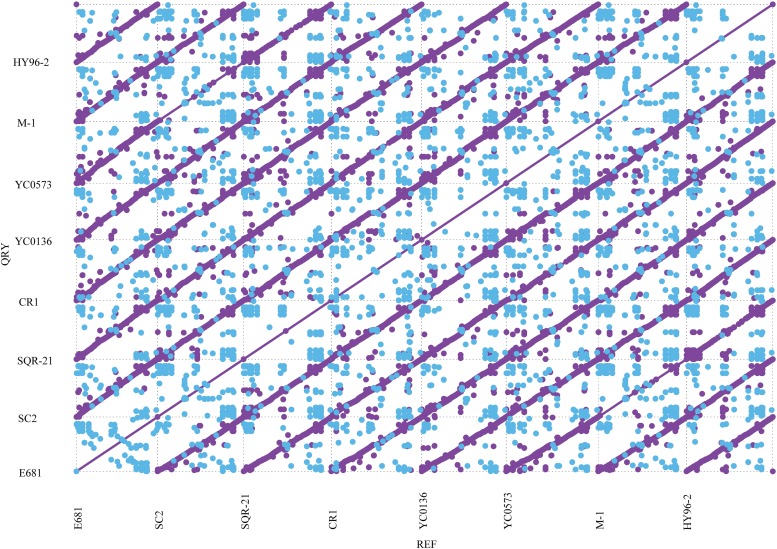
Co-linearity results for strain HY96-2 and the seven other *P. polymyxa* strains. The purple dots represent the forward alignment of the two genome sequences, and the blue dots represent the reverse alignment of the corresponding segments.

The phylogenetic tree indicated that HY96-2 is closest to *P. polymyxa* SQR-21 (**Figure 3**). However, *P. polymyxa* strains M-1 and SC2 were found to exhibit low homologies with strain HY96-2. We compared the 16S rRNA sequence of each *P. polymyxa* strain to calculate the percentage of homology (Supplementary Table S4). The percentage of homology between strain HY96-2 to strains M-1 and SC2 were 97.87 and 97.82%. We also performed a pan-genome analysis to compare HY96-2 and the seven other *P. polymyxa* strains, namely, SC2, E681, YC0136, M-1, SQR-21, CR1, and YC0573. As shown in **Figure 4**, 3382 gene families were found to be involved in the core genome shared by all of the studied strains. In addition, the number of gene families unique to strain HY96-2 was 448, corresponding to 468 genes, which was the highest among all of the analyzed strains (Supplementary Table S5). The annotation revealed that these specific genes encoded a large number of transcriptional regulators, helicase domain proteins, hypothetical proteins, aminotransferases, transposases, drug resistance transporters, and chloramphenicol resistance proteins, etc. The nucleic acid co-linearity results showed that strain HY96-2 has high co-linearity with the seven other *P. polymyxa* strains (**Figure 5**).

### Comparison of Genes Involved in Biofilm Formation

Based on the studies of Yang et al. (2014) and Molinatto et al. (2017), we searched the genomes of strains HY96-2, SC2, and E681 for genes related to biofilm formation and compared their similarities using BLAST. These genes are as follows: the key quorum-sensing gene *luxS*, which affects biofilm formation (Yang et al., 2014); the flagellar motility-related genes, *motA*, *motB*, and *flgM* (Domka et al., 2007; Hu et al., 2011); and the *Bacillus*-based biofilm formation pathway genes, *kinB*, *spo0A*, *spo0F*, *degU*, and *degS*, etc. (Grossman et al., 1992). The results showed that 14 genes involved in biofilm formation were certainly found in the genomes of these three strains. The identities of these genes between HY96-2 and SC2 exceeded 93%, which were higher than those between HY96-2 and E681. In particular, the identities of the genes *motA*, *sfp*, and *kinB* between strain HY96-2 and E681 were below 90% (**Table 3**).

**Table 3:** Comparison of core genes involved in biofilm formation in strain HY96-2, as well as in strains SC2 and E681 (sequence similarity is expressed as a percentage of the nucleotide identity).

Gene name	Locus tag			Product	Identity (%)	
			
	HY96-2	SC2	E681		SC2	E681
*luxS*	C1A50_RS02845	PPSC2_RS31750	PPE_RS02675	*S*-ribosylhomocysteine lyase	97.19	91.99
*Efp*	C1A50_RS14785	PPSC2_RS43880	PPE_RS13870	Elongation factor P	98.57	93.19
*degU*	C1A50_RS22485	PPSC2_RS51810	PPE_RS21415	DNA-binding response regulator	95.99	93.64
*degS*	C1A50_RS22490	PPSC2_RS51815	PPE_RS21420	Histidine kinase	98.71	96.30
*flgM*	C1A50_RS22450	PPSC2_RS51780	PPE_RS21385	Flagellar biosynthesis anti-sigma factor FlgM	97.10	94.57
*motA*	C1A50_RS00510	PPSC2_RS29245	PPE_RS00510	Flagellar motor protein MotA	98.24	88.18
*motB*	C1A50_RS00505	PPSC2_RS29240	PPE_RS00505	Flagellar motor protein MotB	97.22	91.18
*sacB*	C1A50_RS03600	PPSC2_RS32495	PPE_RS03320	Levansucrase	95.00	93.60
*sfp*	C1A50_RS07585	PPSC2_RS36260	PPE_RS07145	4′-phosphopantetheinyl transferase	93.02	86.32
*sigD*	C1A50_RS09600	PPSC2_RS38730	PPE_RS09310	RNA polymerase sigma factor SigD	96.58	94.04
*spo0A*	C1A50_RS14655	PPSC2_RS43750	PPE_RS13740	Sporulation transcription factor spo0A	97.39	93.66
*spo0F*	C1A50_RS00680	PPSC2_RS29415	PPE_RS00675	Sporulation initiation phosphotransferase F	99.46	95.39
*kinB*	C1A50_RS19570	PPSC2_RS48955	PPE_RS18645	Two-component sensor histidine kinase	96.95	89.75
*hfq*	C1A50_RS13605	PPSC2_RS42680	PPE_RS12655	RNA chaperone Hfq	97.53	93.09


**FIGURE 6:**
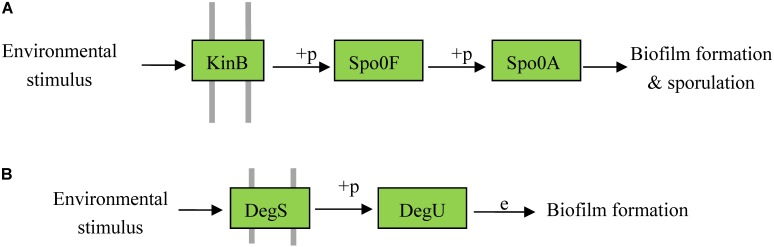
Possible pathways of biofilm formation in *P. polymyxa*. **(A)** Pathway A. **(B)** Pathway B.

To date, no studies have been reported on the biofilm formation pathway in *P. polymyxa*. We explored the possible pathways responsible for biofilm formation in *P. polymyxa* by combining genomes and the information from KEGG database. An important biofilm formation pathway in *Bacillus* is as follows: (1) the sensor histidine kinase KinB (or KinA, KinC, or KinD) phosphorylates its own conserved histidine residue in response to an environmental stimulus; (2) the phosphate group is transferred from the activated KinB to Spo0F; (3) because Spo0F lacks an effector domain, the phosphate group of Spo0F-P is then transferred to the intermediary molecule Spo0B; (4) finally, the receptor domain of the effector Spo0A is phosphorylated by the phosphate group of Spo0B-P and subsequently triggers biofilm formation (Fabert et al., 1999; Jiang et al., 2000). However, no *spo0B* gene was detected in the genomes of the *P. polymyxa* strains. By querying the KEGG database, another pathway involved in sporulation in *B. subtilis* was found, which revealed that Spo0F-P can directly transfer the phosphate group to the effector Spo0A without Spo0B acting as a mediator, and when a high concentration of Spo0A-P accumulates it will trigger the sporulation. Therefore, we propose that a biofilm formation pathway exists in *P. polymyxa* in which KinB autophosphorylates in response to an environmental stimulus and then subsequently phosphorylates the Spo0F response regulator, whereupon the phosphate group is directly transferred to Spo0A to trigger biofilm formation (**Figure 6A**). In addition, *degS* and *degU* genes were detected in the genome of the three *P. polymyxa* strains. It is therefore presumed that another possible biofilm formation pathway in *P. polymyxa* involves activation of the sensor histidine kinase DegS by the external stimulus to phosphorylate the response regulation protein DegU, thereby triggering biofilm formation (**Figure 6B**). This biofilm formation pathway has already been reported in *B. subtilis* (Stanley and Lazazzera, 2005).

### Comparison of Genes/Gene Clusters Involved in Antibiotic Synthesis

The annotation of gene clusters related to secondary metabolite synthesis was performed using the antiSMASH database. For strain HY96-2, 15 gene clusters related to secondary metabolites were retrieved, accounting for 15.77% of the genome; the corresponding values for strains SC2 and E681 were 12 (11.89%) and 11 (9.85%), respectively. Among these three strains, HY96-2 exhibited a greater variety and higher content of secondary metabolites in the genome. The comparison of genes/gene clusters involved in antibiotic synthesis showed that the identities between strains HY96-2 and SC2 were significantly higher than those between strains HY96-2 and E681 (**Table 4**).

**Table 4:** Comparison of gene clusters and core genes involved in antibiotic biosynthesis in strain HY96-2, as well as in strains SC2 and E681.

Antibiotic name	Activity	Locus tag			Type	Identity (%)	
				
		HY96-2	SC2	E681		SC2	E681
Fusaricidin	Broad-spectrum antifungal activity, especially against *Fusarium* sp.; also suppresses G^+^ bacteria (Kajimura and Kaneda, 1995, 1996; Liu et al., 2011)	C1A50_RS00290- C1A50_RS00500	PPSC2_RS29015- PPSC2_RS29230	PPE_RS00290- PPE_RS00500	NRPS	81.54	61.53
Paenilarvins	Broad-spectrum antifungal activity (Sood et al., 2014)	C1A50_RS04650- C1A50_RS04845	PPSC2_RS33500- PPSC2_RS33695	–	TransATPKS– NRPS	81.88	–
Polymyxin	Broad-spectrum antibacterial activity, especially against G^-^ bacteria (Falagas and Kasiakou, 2005; Gounani et al., 2018)	C1A50_RS21330- C1A50_RS21525	PPSC2_RS50695- PPSC2_RS50860	PPE_RS20180- PPE_RS20350	NRPS	63.21	54.94
Polyketide	Suppresses G^-^ bacteria (Chakraborty et al., 2018)	C1A50_RS00805	PPSC2_RS29535	PPE_RS00800	PKS	94.93	90.29
Tridecaptin	Suppresses G^-^ bacteria (Cochrane et al., 2015)	C1A50_RS10840- C1A50_RS11035	PPSC2_RS40010- PPSC2_RS40205	–	NRPS	31.82	–
Bacitracin	Suppresses G^+^ bacteria (Konzl et al., 1997)	C1A50_RS24385	PPSC2_RS53660	PPE_RS23255	NRPS	94.38	90.62
Kalimantacin	Suppresses G^+^ bacteria (Thistlethwaite et al., 2017)	C1A50_RS15500- C1A50_RS15755	PPSC2_RS44500- PPSC2_RS44755	–	TransATPKS– Otherks-NRPS	68.75	–
Mersacidin	Suppresses G^+^ bacteria (Sahl and Bierbaum, 1998)	C1A50_RS07480- C1A50_RS07590	PPSC2_RS36150- PPSC2_RS36265	PPE_RS06970- PPE_RS07075	Lantipeptide	83.54	72.92


Two gene clusters involved in fungicide synthesis (fusaricidins and paenilarvins) as well as six genes or gene clusters involved in bactericide synthesis (polymyxin, tridecaptin, mersacidin, kalimantacin, polyketide, and bacitracin) were found in the genomes of strains HY96-2 and SC2. However, no gene clusters for the biosynthesis of paenilarvins, tridecaptin, or kalimantacin were detected in the E681 genome. For the main antimicrobial agents in *P. polymyxa*, fusaricidins and polymyxin, the comparison revealed that the synthetic gene clusters in strains SC2 and E681 did not exhibit very high similarities with those in strain HY96-2, but the similarities of the corresponding synthetic gene clusters between SC2 and HY96-2 were still higher than those between E681 and HY96-2. The identity of the fusaricidin synthetic gene clusters between SC2 and HY96-2 was 81.54%, whereas that between E681 and HY96-2 was only 61.53%. Similarly, the identity of the polymyxin synthetic gene clusters between SC2 and HY96-2 was 63.21%, whereas that between E681 and HY96-2 was only 54.94%. These variations in the genes and gene clusters involved in antibiotic synthesis between the three strains may explain the differences in their target profiles and efficiency against plant diseases.

### Comparison of Genes Involved in Resistance Inducer Synthesis

Key genes involved in the synthesis of resistance inducers in *P. polymyxa* were retrieved from the KEGG database, and the identities of these genes between HY96-2 and SC2 and E681 were also compared. The results revealed that the key genes for 2,3-butanediol, methanethiol, and isoprene were all found in the genomes of HY96-2, SC2, and E681, with sequence identities exceeding 86%. The identities of the corresponding genes between HY96-2 and SC2 (91.33–97.05%) were significantly higher than those between HY96-2 and E681 (86.83–92.05%) (**Table 5**). This indicates that the three strains can be expected to possess similar abilities to induce plant resistance, although their induction efficiencies may differ owing to the variation in the corresponding genes.

**Table 5:** Comparison of genes involved in the synthesis of resistance inducers in strain HY96-2, as well as in strains SC2 and E681.

Resistance inducer	Gene	Locus tag			Identity (%)	
			
		HY96-2	SC2	E681	SC2	E681
2,3-Butanediol	*alsD*	C1A50_RS10210	PPSC2_RS39445	PPE_RS09950	95.18	90.76
	*ilvN*	C1A50_RS07135	PPSC2_RS35800	PPE_RS06620	95.47	91.98
Methanethiol	*mtnE*	C1A50_RS13950	PPSC2_RS43025	PPE_RS13035	93.23	89.85
	*metH*	C1A50_RS13320	PPSC2_RS42420	PPE_RS12455	95.47	89.74
	*metE*	C1A50_RS23245	PPSC2_RS52550	PPE_RS22160	94.52	88.18
	*mmuM*	C1A50_RS24195	PPSC2_RS53470	PPE_RS23100	94.30	87.13
Isoprene	*idi*	C1A50_RS22765	PPSC2_RS52085	PPE_RS21690	92.55	86.83
	*lytB*	C1A50_RS07370	PPSC2_RS36040	PPE_RS06865	91.33	86.83
	*gcpE*	C1A50_RS18800	PPSC2_RS48225	PPE_RS17845	97.05	91.24
	*ispF*	C1A50_RS21940	PPSC2_RS51265	PPE_RS20840	94.76	89.10
	*ispE*	C1A50_RS00170	PPSC2_RS28895	PPE_RS00170	96.14	92.05


## Discussion

Microbial biopesticides have attracted increasing attention over recent years owing to their effectiveness, environmental friendliness, and safety toward humans and livestock (Berg, 2009). The development of microbial biopesticides in China is also proceeding rapidly and the number of registered and commercially available ones is growing sharply, in line with the Chinese government’s policy of “two reductions” (i.e., reducing the amounts of chemical pesticides and fertilizers used). As an important biocontrol agent, *P. polymyxa* is a relatively novel species and the number of registered products based on this species is growing rapidly^8^. Microbial biopesticides based on *P. polymyxa* have also been produced industrially. However, only a few comprehensive studies of the biocontrol mechanism of *P. polymyxa* at the molecular level have been published to date (Eastman et al., 2014a; Xie et al., 2016). The previous reports concerning the complete genome of *P. polymyxa* predominantly focused on the general features of the genome and analysis of the effect of this species on promoting growth, but rarely involved the analysis of the biocontrol function and mechanism (Kim et al., 2010; Ma et al., 2011; Niu et al., 2011; Eastman et al., 2014b; Li et al., 2014; Liu et al., 2017a,b). In this study we have presented the complete genome of the industrial *P. polymyxa* strain HY96-2, which consists of a 5.75 Mb chromosome. The genome of HY96-2 was compared with those of seven other *P. polymyxa* strains (SC2, E681, YC0136, M-1, SQR-21, CR1, and YC0573). In particular, the biocontrol-related genes and gene clusters involved in the formation of biofilms, antibiotics, and systemic resistance inducers were compared between HY96-2 and the SC2 and E681 strains. The results of this comparison revealed that the genome of strain HY96-2 exhibits some degree of variation. And this finding possibly explained the relationship between genes relating to the biocontrol mechanism and the biocontrol target and efficacy.

According to the phylogenic tree, the homology between strains HY96-2 and E681 is greater than that between strains HY96-2 and SC2. However, comparison of the genes involved in biocontrol mechanism and 16s rRNA (Supplementary Table S4) suggested that strain HY96-2 is more similar to SC2 than E681. SC2 contains a large 0.51 Mb plasmid, whereas HY96-2 and E681 do not. This could be the main reason why the phylogenetic tree suggested that strain HY96-2 is closer to E681 than SC2. Strain SC2 was isolated from rhizosphere of pepper in Guizhou Province, China (latitude 24°37′–29°13′N), and strain HY96-2 was obtained from rhizosphere of tomato in Nanchang, Jiangxi Province, China (latitude 28°16′–28°58′N). Strain E681 was isolated from rhizosphere of winter barley in South Korea (latitude 33°–43°N). The biggest difference between these three strains were the regions and hosts where they were isolated. Strains HY96-2 and SC2 were all isolated from Solanaceae plants, and their geographic locations were the nearest, and they demonstrated the highest homology by comparing the genes related to biocontrol mechanism and 16s rRNA within the three strains. Thus, we speculated the host environment might be one of the reasons of the variation of strains HY96-2, SC2, and E681. Pan-genome analysis revealed that the number of specific gene families in strain HY96-2 was 448, corresponding to 468 genes, which was the highest among the eight *P. polymyxa* strains studied. These unique genes of strain HY96-2 were found to encode transcriptional regulators, helicase domain proteins, aminotransferases, transposases, etc. These functional genes might affect biofilm formation and the production of antibiotics and systemic resistance inducers. Thus, the variation and specialization of these genes could possibly result in the differences in biocontrol targets and efficacy between strain HY96-2 and the other *P. polymyxa* strains.

Biofilm formation is an important trait that has been linked to the colonization ability of biocontrol microorganisms (Ongena and Jacques, 2008). Comparison of the main genes involved in biofilm formation revealed that all 14 of the target genes could be found in strains HY96-2, SC2, and E681, and the similarities of these genes between strains SC2 and HY96-2 were much higher than those between strains HY96-2 and E681. In addition, quorum sensing has been reported to significantly affect biofilm formation in bacteria (Miller and Bassler, 2001). For *B. subtilis*, which can be considered a representative G^+^ biocontrol bacterium, it has been reported that quorum sensing is mainly regulated by the *comA*, *comP*, *comX*, and *comQ* genes (Schneider et al., 2002). These *B. subtilis* quorum-sensing-related genes were deficient in the eight *P. polymyxa* strains studied, although another important quorum-sensing regulatory gene, *luxS*, was observed. *luxS* is a key regulatory gene in quorum sensing mediated by autoinducer 2 (AI-2) (Federle, 2009; Ma et al., 2017), which significantly affects biofilm formation in *P. polymyxa* (this conclusion was obtained in other study by our group, which has not yet been published). In addition, *luxS* was also found to be a key regulatory gene involved in quorum sensing in *P. polymyxa* CR1 (Eastman et al., 2014a). From the above results, it is possible to speculate that all three strains, HY96-2, SC2, and E681, could prevent disease by forming biofilms, but the biofilm formation ability of the three strains might differ owing to the variations in the genes involved in biofilm formation observed in their genomes.

In *B. subtilis*, Spo0A is a key transcriptional regulatory protein that controls the expression of over 100 genes, including those involved in biofilm formation and sporulation. DegU is also a global regulator in *B. subtilis* and controls multiple cellular processes such as competence, motility, and hydrolase secretion, without which biofilm formation would be unsuccessful (Kobayashi, 2007). As confirmed using the KEGG database, the genes related to the biofilm formation pathways regulated by these two key genes were found in all of the three tested *P. polymyxa* strains. Therefore, a possible pathway for biofilm formation in *P. polymyxa* can be preliminarily proposed as follows: in response to an environmental stimulus, KinB phosphorylates the Spo0F response regulator, which subsequently transfers the phosphate group directly to Spo0A to trigger biofilm formation. The main differences in the pathways mentioned above between *P. polymyxa* and *B. subtilis* are that the mediator Spo0B, which transfers the phosphate group between Spo0F and Spo0A, is present in *B. subtilis* but absent from *P. polymyxa*, and that the phosphate group can be directly transferred from Spo0F to Spo0A in *P. polymyxa*. Moreover, another possible pathway for biofilm formation in *P. polymyxa* was deduced, wherein the histidine kinase sensor DegS phosphorylates the response regulator protein DegU to form the biofilm in response to an external stimulus. To date, there have been no reports of the biofilm formation pathway in *P. polymyxa*. Thus, these two possible pathways for biofilm formation in *P. polymyxa* were first deduced and revealed in this paper and found to possess high similarity with those reported in *B. subtilis*.

Comparison of the genes and gene clusters involved in antibiotic synthesis revealed more significant differences between the three *P. polymyxa* strains than those involved in biofilm formation. Strains HY96-2 and SC2 exhibited relatively high homology, whereas strains HY96-2 and E681 had relatively low homology in terms of the genes and gene clusters involved in antibiotic production. The variations in these genes and gene clusters between the three strains might be responsible for the differences in the biocontrol targets and efficacies.

For the control of fungi, as the main antifungal metabolites in *P. polymyxa*, gene clusters for fusaricidins were found in all three of the tested *P. polymyxa* strains. To date, at least ten members of the fusaricidin family have been isolated from *P. polymyxa*, including fusaricidins A–D, LI-F03, LI-F04, LI-F05, LI-F06, LI-F07, and LI-F08 (Liu et al., 2011). Fusaricidins display excellent antifungal activities against many plant pathogenic fungi, especially *F. oxysporum*; fusaricidin B is particularly effective against *Candida albicans* and *Saccharomyces cerevisiae*. Fusaricidins also exhibit excellent germicidal activity against G^+^ bacteria such as *Staphylococcus aureus* (Kajimura and Kaneda, 1995, 1996; Liu et al., 2011). The gene cluster for fusaricidin synthesis in strain HY96-2 showed 81.54% identity with that in strain SC2, which was higher than that for strain E681 (61.53% identity). Fusaricidin A was the main fusaricidin found in strain HY96-2 (Liu et al., 2011). Choi et al. (2008) discovered that the *fusA* gene in E681 plays an important role in fusaricidin biosynthesis, and the inactivation of this gene led to the complete loss of antifungal activity against *F. oxysporum*; moreover, *fusA* can produce more than one kind of fusaricidin. Though Mikkola et al. (2017) mention that fusaricidin A and B possess toxicity to mammalian cells at a certain concentration, the results of animal and environmental toxicology tests of the products with living cells of *P. polymyxa* HY96-2 showed that the animal and environmental toxicities of the products were slight and low, respectively, at the lowest level of toxicity in the corresponding test (Supplementary Figures S2, S3). Thus, we considered that as long as fusaricidins were not purified and developed as pesticide directly, microbial pesticides with living cells of *P. polymyxa* are environmentally friendly and safe toward animal. In addition, the gene clusters for paenilarvins, a class of iturin-like compounds with broad-spectrum antifungal activity, were also found in strains HY96-2 and SC2 but not in E681. The identity of the paenilarvin biosynthetic gene clusters between HY96-2 and SC2 was 81.88%. All three strains were reported to suppress the plant pathogenic fungus *F. oxysporum* and *B. cinerea*. Besides *F. oxysporum* and *B. cinerea*, the reported control targets of the three strains were different (**Table 1**). Strains HY96-2 and E681 were also found to suppress *R. solani*. The different biocontrol targets of these three strains may be attributable to the variation in their gene clusters responsible for the synthesis of antifungal metabolites.

For the control of bacteria, many differences were also observed between the three strains in terms of the genes or gene clusters involved in the synthesis of antibacterial metabolites. Using the antiSMASH database, six genes or gene clusters related to antibiotic synthesis were found in HY96-2 and SC2, including those involved in the biosynthesis of polymyxin, tridecaptin, polyketide, mersacidin, kalimantacin, and bacitracin. In contrast, only four were detected in strain E681, as the genes or gene clusters for tridecaptin and kalimantacin synthesis were absent. Among the six antibiotics, polymyxin, tridecaptin, and polyketide are able to suppress G^-^ bacteria (Cochrane et al., 2015; Chakraborty et al., 2018; Gounani et al., 2018), whereas mersacidin, kalimantacin, and bacitracin show activity against G^+^ bacteria (Konzl et al., 1997; Sahl and Bierbaum, 1998; Thistlethwaite et al., 2017). The identities of the genes or gene clusters related to the six antibiotics were clearly higher between strains HY96-2 and SC2 than between strains HY96-2 and E681. All three of the *P. polymyxa* strains were found to contain polymyxin-related gene clusters. Polymyxin is another key antibiotic in *P. polymyxa* and possesses broad-spectrum antibacterial activity, especially against G^-^ bacteria (Falagas and Kasiakou, 2005). However, the polymyxin-related gene clusters in strains SC2 and E681 displayed low identities with that in strain HY96-2 of only 63.21 and 54.94%, respectively. Bacterial plant diseases are mainly caused by G^-^ bacteria, such as those belonging to the genera *Ralstonia* (Liu et al., 2017c), *Erwinia* (Bell et al., 2004), *Pseudomonas* (Wang et al., 2018), and *Xanthomonas* (Islam et al., 2018). Strain HY96-2 has been found to effectively control many G^-^ bacteria plant diseases in the field (**Table 1**), such as bacterial wilt, a soil-borne disease caused by *R. solanacearum* that has been referred to as “plant cancer,” and angular leaf spot on cucumbers, a leaf disease caused by *P. syringae*. In the field, the biocontrol efficacy of 10^9^ CFU/g *P. polymyxa* wettable powder (PPWP, developed with strain HY96-2) on tomato bacterial wilt was found to reach 91.03% with a dosage of 10.8 kg/hectare. Moreover, the biocontrol efficacy of PPWP against angular leaf spot on cucumbers reached 81.08% with 300-fold dilution, which was significantly higher than that obtained for a chemical pesticide (Supplementary Table S6). In contrast to strain HY96-2, neither strain E681 nor strain SC2 has found application as a bactericide. Strain E681 was only reported to inhibit the growth of the G^-^ plant pathogenic bacterium *P. syringae* and the G^-^ human pathogenic bacterium *Escherichia coli* (**Table 1**), while the detailed and definitive antibacterial spectrum of strain SC2 has not been reported. It can be deduced that the variations of the genes and gene clusters involved in antibacterial metabolite synthesis between these three strains might be responsible for the differences in their antibacterial spectra and control efficacy.

Comparison of the key genes involved in systemic resistance inducer production revealed that all three of the tested strains contained the key genes related to volatile organic compounds (2,3-butanediol, methanethiol, and isoprene). The genes responsible for these inducers in strains SC2 and HY96-2 exhibited 91.33–97.05% identity, which is higher than the 86.83–92.05% identity observed between strains E681 and HY96-2. It can be deduced that the three strains can induce similar systemic resistance in plants but with varying effectiveness owing to the variations in the related genes.

In summary, in this study we determined the complete genome sequence of *P. polymyxa* strain HY96-2 and performed a comparative genomic analysis between various *P. polymyxa* strains. Based on this comparison, especially in terms of biofilm formation, antibiotic synthesis, and systemic resistance inducer production, the biocontrol mechanisms of *P. polymyxa* strain HY96-2 were determined at the molecular level. These mechanisms can be deduced as follows: (1) biofilm formation to prevent infection of the plant; (2) synthesis of fusaricidins and paenilarvins to protect against fungal plant pathogens, and the secretion of polymyxin, tridecaptin, and polyketide antibiotics with activity against G^-^ bacteria plant pathogens and the production of mersacidin, kalimantacin, and bacitracin with activity against G^+^ bacteria plant pathogens; (3) induction of the systemic resistance of plants via inducing volatile organic compounds, including 2,3-butanediol, methanethiol, and isoprene, etc. The differences between the various *P. polymyxa* strains in terms of the control targets and efficacies might be attributable to the variations in the genes or gene clusters responsible for these three aspects of the biocontrol mechanism. The goal of this study was to provide a scientific basis for the further optimization of microbial biopesticides based on *P. polymyxa* strain HY96-2 in terms of field application and quality standards. For example, to develop a biopesticide based on strain HY96-2 for specifically controlling plant diseases caused by G^-^ bacteria, the levels of polymyxin in the product could be increased by altering the fermentation conditions or genetically modifying the producing strain. This study may also serve as a reference for future investigations into the differences in biocontrol targets and efficacy between different biocontrol agents.

## Author Contributions

YCL, YC, and YGL experimental design and authorship. YCL, YC, JY, ZZ, and QL experiments and data analysis. YCL, YC, YGL, and DZ manuscript revision. All authors read and approved the final manuscript.

## Conflict of Interest Statement

The authors declare that the research was conducted in the absence of any commercial or financial relationships that could be construed as a potential conflict of interest.

## Footnotes

1 https://www.epa.gov/tsca-inventory/list-substances-reported-under-tsca-inventory-notification-active-inactive-rule

2 http://www.icama.org.cn/hysj/index.jhtml

3 http://www.ec.gc.ca/ese-ees/9F3909AA-3024-4BBD-AC9E-2EB681ED1BBD/FSAR_Paenibacillus%20Polymyxa_EN.pdf

4 http://www.vicbioinformatics.com/software.barrnap.shtml

5 http://mafft.cbrc.jp/alignment/software/

6 http://molevol.cmima.csic.es/castresana/Gblocks.html

7 http://www.genome.jp/kegg/pathway.html

8 http://www.icama.org.cn/fwb/index.jhtml
