# Supplementary material for: Complete Genome Sequence of Industrial Biocontrol Strain Paenibacillus polymyxa HY96-2 and Further Analysis of Its Biocontrol Mechanism
Source: Front Microbiol. 2018 Jul 12;9:1520. doi: 10.3389/fmicb.2018.01520 (PMC6052121; doi:10.3389/fmicb.2018.01520)
Supplement: Supplementary file 1 [file Data_Sheet_1.pdf]

Complete Genome Sequence of Industrial Biocontrol Strain *Paenibacillus polymyxa* HY96-2 and Further Analysis of Its Biocontrol Mechanism

Yuanchan Luo, Yuejuan Cheng, Jincui Yi, Zhijun Zhang, Qian Luo, Daojing Zhang*, and Yuanguang Li*

* Co-corresponding authors: Yuanguang Li, ygli@ecust.edu.cn; Daojing Zhang, djz@ecust.edu.cn.

Supplementary Materials

Content

Supplementary Figure S1: Distribution diagram of GO functional annotation in the *P. polymyxa* strain HY96-2 genome.

Supplementary Figure S2: The results of animal toxicology test of product of *P. polymyxa* strain HY96-2.

Supplementary Figure S3: The results of environmental toxicology and safety evaluation test of product of *P. polymyxa* strain HY96-2.

Supplementary Table S1: Genomic islands in *P. polymyxa* strain HY96-2

Supplementary Table S2: CRISPR results for the genome of *P. polymyxa* strain HY96-2

Supplementary Table S3: Number of genes in each of the COG functional categories for *P. polymyxa* strain HY96-2

Supplementary Table S4: Homology of 16s rRNA between HY96-2 and other strains of *P. polymyxa*

Supplementary Table S5: Number of specific gene families and genes in each of the *P. polymyxa* strains

Supplementary Table S6: Biocontrol efficacies of *P. polymyxa* wettable powder against bacterial plant diseases in field tests

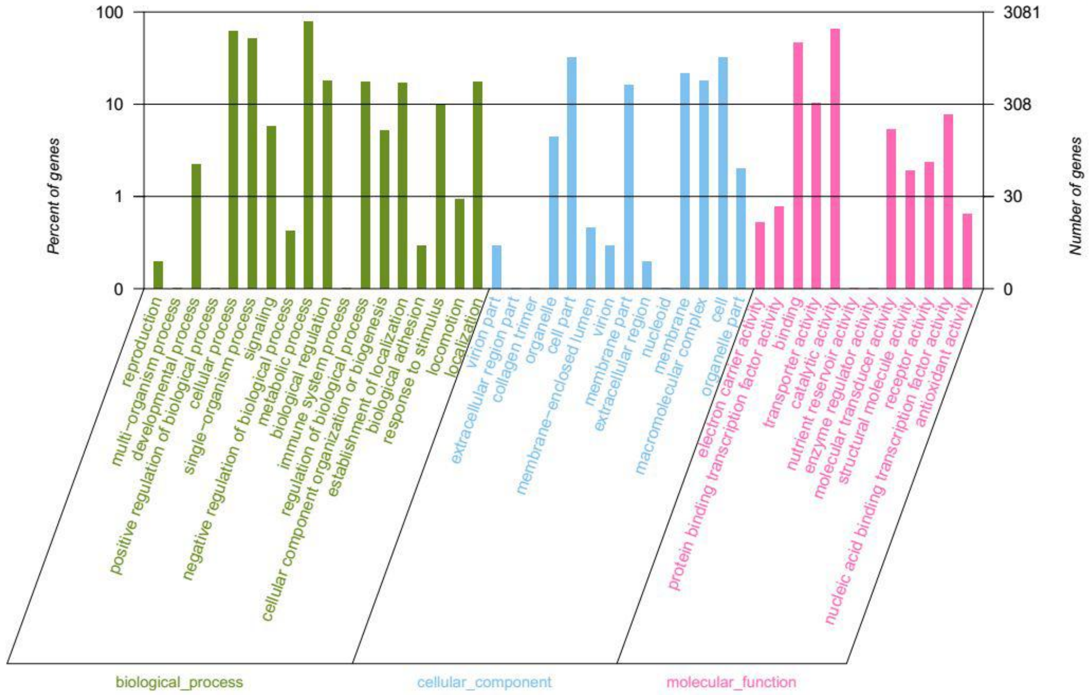

Supplementary Figure S1: Distribution diagram of GO functional annotation in the *P. polymyxa* strain HY96-2 genome.

Supplementary Figure S2: The results of animal toxicology test of product of *P. polymyxa* strain HY96-2.

Summary of the results of animal toxicology test of 5 billion cfu/g technical material of *P. polymyxa* HY96-2

The tests were conducted by School of Public Health, Fudan University, China.

① Acute skin toxicity test in rats: LD_{50}>5000mg/kg, slight toxicity;
② Acute oral toxicity test in rats: LD_{50}>5000mg/kg, slight toxicity;
③ Acute inhalation toxicity test in rats： LD_{50}>5000mg/m^3, slight toxicity;
④ Eye irritation test in rabbits: no irritation;
⑤ Guinea-pig skin sensitization test: weak sensitizer
⑥ Pathogenicity test in mice: no pathogenicity

Conclusion: 5 billion cfu/g technical materia of *P. polymyxa* HY96-2 was a slightly toxic pesticide (slight toxicity is at the lowest level of toxicity in the test.).

FUDAN UNIVERSITY 1905

复旦大学公共卫生学院毒理学教研室毒性测试报告

"50 亿 CFU/g 多粘类芽孢杆菌原药"
毒理学试验报告

备案号： DL0903310210438
实验项目： 大鼠急性经口毒性试验
大鼠急性经皮毒性试验
大鼠急性吸入试验
家兔眼刺激试验
豚鼠皮肤致敏试验
测试样品： 50 亿 CFU/g 多粘类芽孢杆菌原药
送检单位： 浙江省桐庐汇丰生物化工有限公司
样品接受日期： 2009 年 3 月 27 日

复旦大学公共卫生学院毒理学教研室
2009 年 5 月 27 日

"50 亿 CFU/g 多粘类芽孢杆菌原药"
大鼠急性经口毒性试验报告

测 试 物　"50 亿 CFU/g 多粘类芽孢杆菌原药"
送检单位　浙江省桐庐汇丰生物化工有限公司
封样单位　浙-2009-052
送检日期　2009 年 3 月 27 日
测试日期　2009 年 3 月 31 日～2009 年 4 月 14 日
样品性状　浅棕色粉末
测试方法　按 GB 15670-1995 中华人民共和国国家标准"农药登记毒理学试验方法"进行
样品处理　样品用蒸馏水配制成试验用液
实验动物　健康 SD 成年大鼠，20 只，雌雄各半，体重在 180～200g 范围内，由复旦大学实验动物部提供
动物分组　实验设 1 个剂量组，每组动物 10 只，雌雄各半，剂量为 5000mg/kg
操作步骤　动物给药前隔夜禁食（15 小时），称重后，按 100g 体重给 1ml 灌胃量给药一次，然后观察并记录动物的急性中毒症状和死亡情况，连续观察二周
试验结果：染毒后动物出现体重下降中毒症状、未出现死亡
结果评价　按农药的急性经口毒性分级标准进行判定
结　　论　"50 亿 CFU/g 多粘类芽孢杆菌原药"的急性经口毒性试验，雄性、雌性 LD_{50} 均大于 5000mg/kg，属微毒类农药。

报告单位：复旦大学公共卫生学院毒理学教研室
测试者：顾锡安、周袁芬
审核者：周志俊

二〇〇九年五月二十七日

复旦大学公共卫生学院毒理学教研室毒性测试报告

"50 亿 CFU/g 多粘类芽孢杆菌原药"
大鼠急性经皮毒性试验报告

测 试 物　"50 亿 CFU/g 多粘类芽孢杆菌原药"
送检单位　浙江省桐庐汇丰生物化工有限公司
封样单位　浙-2009-052
送检日期　2009 年 3 月 27 日
测试日期　2009 年 4 月 7 日～2009 年 4 月 21 日
样品性状　浅棕色粉末
测试方法　按 GB 15670-1995 中华人民共和国国家标准"农药登记毒理学试验方法"进行
样品处理　样品用蒸馏水配制成试验用液
实验动物　健康 SD 成年大鼠，20 只，雌雄各半，体重在 200～300g 范围内，由复旦大学实验动物部提供
动物分组　动物设 1 个剂量组，每组动物 10 只，雌雄各半。剂量为 5000mg/kg
操作步骤　动物给药前 24 小时背部去毛，面积为 4cm×5cm。称重后，进行定量敷药，每 100g 体重 0.5ml，然后覆盖盒式罩，以胶布固定。涂药持续时间为 4 小时，随后用水洗去皮肤上残留的农药，观察记录动物皮肤的局部反应和急性中毒反应，观察时间为 2 周
试验结果：染毒后动物未出现明显中毒症状、体征和死亡
结果评价　按农药的急性经皮毒性分级标准进行判定
结　　论　"50 亿 CFU/g 多粘类芽孢杆菌原药"的大鼠急性经皮毒性试验，雄性、雌性 LD_{50} 均大于 5000mg/kg，属微毒类农药。

报告单位：复旦大学公共卫生学院毒理学教研室
测试者：顾锡安、周袁芬
审核者：周志俊

二〇〇九年五月二十七日

“50 亿 CFU/g 多粘类芽孢杆菌原药”
大鼠急性吸入毒性试验报告

测 试 物	“50 亿 CFU/g 多粘类芽孢杆菌原药”
送检单位	浙江省桐庐汇丰生物化工有限公司
封样单位	浙-2009-052
送检日期	2009 年 3 月 27 日
测试日期	2009 年 4 月 8 日～2009 年 4 月 22 日
样品性状	浅棕色粉末
测试方法	按 GB 15670-1995 中华人民共和国国家标准“农药登记毒理学试验方法”进行
样品处理	样品直接称量用于试验
实验动物	健康 SD 成年大鼠，20 只，雌雄各半，体重在 180～200g 范围内，由复旦大学实验动物部提供
动物分组	动物设一个剂量组，共 20 只，雌雄各半，剂量为 $5000mg/m^3$
操作步骤	静式染毒，50 升的染毒柜 4 只，每只染毒柜中放大鼠 5 只。将试验用液，称 250mg 放入染毒柜中，样品直接放入底部。将上下放入小型电风扇。再将动物放入染毒柜，密闭。染毒 2 小时，取出动物。观察其反应，连续观察 2 周
试验结果：	染毒大鼠没有出现异常
结果评价	按农药的急性毒性分级标准进行判定
结　　论	“50 亿 CFU/g 多粘类芽孢杆菌原药”的大鼠吸入毒性试验，雄性、雌性的 LC_{50} 均大于 $5000mg/m^3$，属微毒类农药。

报告单位：复旦大学公共卫生学院毒理学教研室
测试者：　顾锡芬　周袁芬
审核者：　周志俊
二○○九年五月二十七日

“50 亿 CFU/g 多粘类芽孢杆菌原药”
家兔眼刺激试验测试报告

测 试 物	“50 亿 CFU/g 多粘类芽孢杆菌原药”
送检单位	浙江省桐庐汇丰生物化工有限公司
封样单位	浙-2009-052
送检日期	2009 年 3 月 27 日
测试日期	2009 年 3 月 31 日～2009 年 4 月 7 日
样品性状	浅棕色粉末
测试方法	按 GB 15670-1995 中华人民共和国国家标准“农药登记毒理学试验方法”进行
样品处理	样品直接用于试验
试验动物	健康新西兰白兔，4 只，双眼正常，体重在 2～3kg 范围内，由复旦大学实验动物部提供
操作步骤	将左侧下眼睑轻轻下拉，将试验用液 100mg 直接滴入动物眼结膜囊内，闭合眼睑约 1 分钟。然后分别在 1 小时、24 小时、72 小时、4 天和 7 天观察动物结膜、角膜、虹膜等眼睛其它部位受损情况
试验结果	见附表 1（另页）
结果评价	每只动物试验结果按眼损伤程度评分表评分，最后按眼睛刺激性分级标准进行判定
结　　论	“50 亿 CFU/g 多粘类芽孢杆菌原药”在对家兔眼刺激试验中，表现为无刺激性。

报告单位：复旦大学公共卫生学院毒理学教研室
测试者：　顾锡芬　周袁芬
审核者：　周志俊
二○○九年五月二十七日

"50 亿 CFU/g 多粘类芽孢杆菌原药"

豚鼠致敏试验报告

**测 试 物**	"50 亿 CFU/g 多粘类芽孢杆菌原药"
**送检单位**	浙江省桐庐汇丰生物化工有限公司
**封样单位**	浙-2009-052
**送检日期**	2009 年 3 月 27 日
**测试日期**	2009 年 3 月 31 日～2009 年 5 月 26 日
**样品性状**	浅棕色粉末
**测试方法**	按 GB 15670-1995 中华人民共和国国家标准"农药登记毒理学实验方法"进行
**样品处理**	样品用蒸馏水配制成试验用液
**实验动物**	健康成年豚鼠，42 只，由复旦大学实验动物部提供
**动物分组**	动物分为 3 组，每组 14 只（雌 7，雄 7）。分别为阴性对照（蒸馏水）组、实验组、阳性对照（二硝基氯苯）组
**操作步骤**	实验前 24 小时动物背部左侧剃毛（3cm × 3cm）。将"50 亿 CFU/g 多粘类芽孢杆菌原药"（称 2g 加 2ml 蒸馏水）、阳性对照（0.5%）和蒸馏水分 14 份涂在 2cm×2cm 的滤纸上，敷贴在剃毛区，固定，6 小时后冲洗。在第 7 天和第 14 天分别重复一次<br>在开始实验（首次涂药）后第 27 天将实验组和阳性对照组动物背部右侧剃毛（2cm × 2cm），次日（第 28 天）涂敷"50 亿 CFU/g 多粘类芽孢杆菌原药"（称 1.5g 加 2ml 蒸馏水）和阳性对照（0.2%）各 0.1ml，固定，6 小时后冲洗。观察至第 42 天。第 43 天实验组动物再涂"50 亿 CFU/g 多粘类芽孢杆菌原药"一次，观察至第 56 天
**试验结果**	阴性对照组和实验组在实验过程中，未发现任何皮肤异常，阳性对照组动物皮肤均有改变
**结果评价**	按农药的皮肤变态（致敏）反应分级标准进行判定
**结　　论**	"50 亿 CFU/g 多粘类芽孢杆菌原药"的豚鼠致敏试验，不引起豚鼠皮肤的改变，属弱致敏物。

报告单位：复旦大学公共卫生学院毒理学教研室
测试者：顾锡安 周袁芬
审核者：周志俊

二〇〇九年五月二十七日

Supplementary Figure S3: The results of environmental toxicology and safety evaluation test of product of *P. polymyxa* strain HY96-2.

Summary of the results of environmental toxicology and safety evaluation test of 5 billion cfu/g technical material of *P. polymyxa* HY96-2

The tests were conducted by Institute of Pesticide and Environmental Toxicology, Zhejiang University, China.

① Quail: LD_{50}（7d）> 1000 mg/kg avoirdupois;
② Bee: LC_{50}（48h）> 2000 mg/L, LD50（48h）> 100 μg/bee, low toxicity;
③ Silkworm: LC_{50}（96h）> 2000 mg/L, low toxicity;
④ Zebrafish: LC_{50}（96h）>100 mg/L, low toxicity;
⑤ Green algae: EC_{50}（72h）= 14.5 mg/L, low toxicity;
⑥ Daphnia magna: LC_{50}（48h）> 200 mg/L, low toxicity.

Conclusion: 5 billion cfu/g technical materiaof *P. polymyxa* HY96-2 was a low toxic pesticide, and safe to environment (low toxicity is at the lowest level of toxicity in the test.).

试验委托单位

浙江省桐庐汇丰生物化工有限公司

试验项目

50 亿 CFU/g 多粘类芽孢杆菌原药
对环境生物毒性试验

备案号：HD0903270070189

试验研究号：2009-T-137

项目负责人：　李少南

报告编写人：　魏方林

报告完成日期

2009 年 8 月 14 日

浙江大学农药与环境毒理研究所

地址：浙江省杭州市凯旋路 268 号

邮编：310029　电话：（0571）86430193　传真：（0571）86971220

试验责任者声明

试验项目：　50 亿 CFU/g 多粘类芽孢杆菌原药对环境生物毒性试验

试验研究号：　2009-T-137

本试验按照中华人民共和国国家环保局《化学农药环境安全评价试验准则》的规定在本研究所计量论证实验条件下进行。试验过程严格按照试验计划书进行，本报告真实而正确反映了试验结果。

试验结果仅对来样负责。

项目负责人（签名）[illegible]　09年 8月14日

报告编写人（签名）[illegible]　09年 8月 14日

实验室负责人（签名）[illegible]　09年 8月14日

试验质量保证书

试验项目：　50 亿 CFU/g 多粘类芽孢杆菌原药对环境生物毒性试验

试验研究号：　2009-T-137

本试验严格按照试验计划书及有关试验方法进行，本报告内容准确地反映了该试验的原始数据，特此证明。

QUA 负责人（签名）[illegible]　2009 年 8 月 24 日

5　结论

表 15　50 亿 CFU/g 多粘类芽孢杆菌原药对非靶生物毒性结果汇总

供试生物	考察指标	测定结果	毒性等级[a]
鹌鹑	LD_{50}（7d）	>1000mg/(kg av.)	低毒
蜜蜂	LC_{50}（48h）	>2000mg/L	低毒
蜜蜂	LD_{50}（48h）	>100μg/bee	低毒
家蚕	LC_{50}（96h）	>2000mg/L	低毒
斑马鱼	LC_{50}（96h）	>100mg/L	低毒
绿藻	EC_{50}（72h）	14.5mg/L	低毒
大型溞	LC_{50}（48h）	>200mg/L	低毒

注：1　"av."为"avoirdupois"缩写，表示"体重"。

2　[a] 依据我国目前采用的农药对环境生物毒性分级标准。

签发人（签名）：

浙江大学农药与环境毒理研究所

2009 年 8 月 14 日

Supplementary Table S1: Genomic islands in *P. polymyxa* strain HY96-2

No.	Start	End	Length
1	556582	562227	5645
2	1277208	1291135	13927
3	2377804	2384236	6432
4	2469151	2507664	38513
5	2592825	2598206	5381
6	2783512	2885231	101719
7	2859583	2863998	4415
8	2875334	2883966	8632
9	3069005	3073460	4455
10	3940617	3949287	8670
11	3955813	3967619	11806
12	3968586	3975853	7267
13	3988233	3994070	5837
14	4929857	4971440	41583
15	5671663	5699257	27594
16	5686148	5693277	7129

Supplementary Table S2: CRISPR results for the genome of *P. polymyxa* strain HY96-2

Method	Start	End	Description
CRISPRFinder	4753030	4753122	Putative

Supplementary Table S3: Number of genes in each of the COG functional categories for *P. polymyxa* strain HY96-2

Functional_categories	Number of genes	Percentage (%)
Translation, ribosomal structure and biogenesis	161	3.09
Transcription	228	4.38
Replication, recombination and repair	111	2.13
Cell cycle control, cell division, chromosome partitioning	23	0.44
Cell wall/membrane/envelope biogenesis	113	2.17
Cell motility	36	0.69
Posttranslational modification, protein turnover, chaperones	85	1.63
Signal transduction mechanisms	135	2.59
Intracellular trafficking, secretion, and vesicular transport	19	0.36
Defense mechanisms	51	0.98
Energy production and conversion	145	2.78
Amino acid transport and metabolism	245	4.71
Nucleotide transport and metabolism	80	1.54
Carbohydrate transport and metabolism	328	6.30
Coenzyme transport and metabolism	96	1.84
Lipid transport and metabolism	80	1.54
Inorganic ion transport and metabolism	208	3.99
Secondary metabolite biosynthesis, transport and catabolism	40	0.77
General function prediction only	202	3.88
Function unknown	219	4.21

Supplementary Table S4: Homology of 16s rRNA between HY96-2 and other strains of *P. polymyxa*

Strain	Resource	Percentage of homology of 16s rRNA with HY96-2 （%）
SC2	Pepper rhizosphere	97.82
E681	Winter barley rhizosphere	97.38
M-1	Wheat rhizosphere	97.87
SQR-21	Melon rhizosphere	98.84
CR1	Degraded corn straw	96.83
YC0136	Tobacco rhizosphere	97.44
YC0573	Tobacco rhizosphere	97.51

Supplementary Table S5: Number of specific gene families and genes in each of the *P. polymyxa* strains

Strain	Number of families	Number of genes
HY96-2	448	468
SC2	58	58
E681	383	395
SQR-21	279	285
M-1	186	196
CR1	216	224
YC0573	174	176
YC0136	213	216

Supplementary Table S6: Biocontrol efficacies of *P. polymyxa* wettable powder against bacterial plant diseases in field tests

Treatment	Biocontrol efficacy[a]	
	Against *R. solanacearum*	Against *P. syringae*
Agent I[b]	91.03±0.71 aA	81.08±3.32 aA
Agent II[b]	83.48±3.22 bB	74.90±2.42 bB
Agent III[b]	73.13±4.54 cC	68.20±1.76 cC
Positive control[c]	66.13±3.59 dC	76.25±1.63 bAB

[a]The two field tests were carried out in Hainan Province, China. The data for the biocontrol efficacies are expressed as the mean ± standard deviation. The SPSS software (version 17.0) was used for the statistical analysis, and the results of Duncan's multiple range test at p=0.05 and p=0.01 are expressed by the lowercase and capital letters, respectively, following each entry. Different lowercase or capital letters following each entry indicate a statistically significant difference between the data in the corresponding confidence level.

[b]The dosages of *P. polymyxa* strain HY96-2 agents I, II, and III used against *R. solanacearum* on tomato were 10^9 CFU/g *P. polymyxa* wettable powder (PPWP) at 10.2, 8.4, and 6.6 kg/hectare, respectively. The dilutions of *P. polymyxa* strain HY96-2 agents I, II, and III used against *P. syringae* on cucumber were 10^9 billion CFU/g PPWP diluted by 300, 600, and 900 times, respectively.

[c]Positive controls against *R. solanacearum* and *P. syringae* were 10^7 CFU/g *P. Polymyxa* granulae subtilae at dosages of 37.5 kg/hectare and 77% Kocide wettable powder at 500 times dilution, respectively.
